# Integrating Smart Technology Into Health Promotion for Older Adults in Taiwan: Managing Cardiovascular Risks

**DOI:** 10.1093/geroni/igaf122.3969

**Published:** 2025-12-31

**Authors:** Jen-Yu Wang, Li-Hui Lin

**Affiliations:** National Chung Cheng University, Hsinchu, Taiwan (Republic of China); National Chung Cheng University, Chiayi, Minxiong, Taiwan (Republic of China)

## Abstract

As Taiwan rapidly transitions into a super-aged society, cardiovascular disease has become a critical health concern for older adults. This study investigates the integration of smart technology into an active aging health promotion program and its impact on cardiovascular risk management and learning motivation. Grounded in achievement motivation theory, the research employs a quasi-experimental design with qualitative inquiry to evaluate both physiological outcomes and participants’ learning experiences. A total of 30 older adults were recruited and assigned to either an experimental group, which received a 8-week program integrating smart technology (e.g., heart spectrum analyzer) with YSD Therapy massage, or a control group, which participated in traditional exercise sessions. Cardiovascular indicators—including blood pressure, heart rate, and heart spectrum indices (I1, I2, I3)—were measured before and after the intervention. Qualitative data from questionnaires and semi-structured interviews were analyzed using Braun and Clarke’s six-phase thematic analysis to identify themes related to motivation, participation, and health self-management. Results showed that the experimental group demonstrated greater improvements in cardiovascular stability, especially in reducing arrhythmia and coronary risk, compared to the control group. Qualitative findings indicated that smart technology enhanced health data visualization, strengthened participants’ sense of control, and fostered stronger motivation to sustain health behaviors. This study demonstrates the feasibility and value of integrating smart technology with traditional health promotion practices. It provides empirical evidence for education-based interventions that improve cardiovascular outcomes while empowering older adults’ motivation, agency, and long-term participation.

